# Case of Congenital Cephalocele

**Published:** 1856-08

**Authors:** W. A. Peck

**Affiliations:** Berwick, Pa.


### MEDICAL EXAMINER.

NEW SERIES.—NO. XL.—AUGUST, 1856.

### ORIGINAL COMMUNICATIONS.

*Case of Congenital Cephalocele.* By W. A. Peck, M. D., Berwick, Pa.

The following case, which I have denominated *cephalocele* in its generic signification, occurred in the practice of Dr. J. A. Wilson.

On the 15th of June, the Doctor was called to Mrs. S., to attend her during her first parturition. Her gestation was of the normal term ; labor advanced as usual up to the rupture of the membranes, at which time an examination was made, which occasioned no little embarrassment concerning the nature of the presenting part. An elastic, soft, and deeply fissured surface presented, which was at first supposed to be the breech, but upon further examination, none of the genitals, or other characteristics of this presentation could be detected. The nature of the part remained a profound puzzle until the birth of the *caput* solved the mystery. The tumor presented, followed by the vertex, and the child was delivered as in normal labor. The child was a female, of the usual size and conformation, save the abnormities of the head. Its physiognomy is very well represented by the outlines of Dr. Rohrer's case, as given by Dr. Meigs in his work on Obstetrics, (page 221,) though somewhat exaggerated. The form of the cranium was conical, with the frontal region somewhat flattened, and the occipital bone quite protuberant ; though, as a whole, symmetrical, and of the usual size. This shape of the cranium, with an imperfectly developed state of the inferior maxilla and prominent malar bones, presented a peculiar vacant, idiotic expression. The cranial bones were completely ossified and firm; suture complete; and fontanelles closed, or nearly so.

The integuments presented at first their normal temperature and color; vessels full, with occasional pulsation; and the scalp thinly covered with hair, except on the most exposed portions of the tumor. The tumor was about the size of the fœtal head at "term;" in its general contour spherical, and about equally divided on the surface by a transverse fissure, of an inch in depth. Its attachment was by a pedicle, of about two inches in diameter, whose centre occupied the occipital protuberance. The upper margin of the pedicle extended to the lambdoidal suture, and its inferior attachment to the inferior semi-circular ridge of the occipital bone. The skin covering the tumor presented its normal appearance near the pedicle, and in the fissure, which shaded off, however, into an attenuated, transparent structure, through which could be clearly recognized large patches of capillary erectile tissue, and plexuses of dilated veins. In consistence, the tumor was quite soft, elastic and distinctly fluctuating. It was loosely attached to the skin, as also to the cranium. Its softness enabled me to demonstrate the continuity of the occipital bone under the pedicle, by inserting my fingers from either side, until the whole tract was explored. By firm and constant pressure, which had the effect of awakening the child from its usual somnolent state, its size could be very much reduced. When thus reduced, pulsation was but slight, but fluctuation more palpable. Long continued compression of the carotids also had the effect of reducing its size and corrugating the skin on its surface. When in its ordinary state of tension, a venous murmur was very perceptible.

This is the result of my physical examination, six days after the child's birth; and I am assured by the Doctor, that this accurately defines its previous history. At this time, the transverse diameter was 4 inches, vertical about 5 inches, and circumference about 13 inches; though these measurements were variable at different times of the day, owing to the different states of the circulatory system, or at least were coincident with them.

A few hours after birth, the child was seized with a spasm, which lasted about twenty-four hours, since which time nothing of the kind manifested itself until the day of its death. It would receive from one to two teaspoonfuls of nourishment at a time with facility, after which, fluids would be ejected from the mouth without deglutition. Its bowels were disposed to be costive, though not enough so to demand much medication. However, the skin became very much jaundiced, and the urine scanty and deeply tinged with bile. The child now became lethargic, with an entire absence of voluntary action of any kind.

The skin of the general surface, in the morning was cold ; this was succeeded by ordinary heat during the middle of the day, and in turn in the evening by high fever. When the general surface was cold, the tumor was very much reduced in size, and its investing integument corrugated. When natural temperature succeeded, the tumor presented the character above described ; but when fever set in, there was a strong determination of blood to the head; the face, scalp, and eyes were deeply suffused ; the arteries were throbbing ; veins distended, and the tumor greatly enlarged ; becoming more firm, less fluctuating, with an increased pulsation and venous murmur, and the skin shining and transparent. The child would now be wakeful. These exacerbations of fever passed off with copious perspiration during the last two days of its existence. During the same period, and at the time of the chill, the left arm, side and leg became livid, and spasmodically affected, without any concurrent action of the right side. This was attributed to an ulcerative abrasion of the skin on the left side of the neck, which, as it merely affected the skin, could afford no adequate explanation of the phenomena. During this time, the tumor was undergoing very important changes. Its growth was rapid ; so much so that at the day of its death (then twelve days old), its several diameters were one half greater than those above given.

Through the importunities of the friends, Dr. W. was induced to puncture the tumor with a lancet. This was followed by profuse arterial hemorrhage, which persisted, in spite of styptic applications, for two days. On examination I discovered that the site of the wound was *in a large tract of cutaneous erectile tissue.* The hemorrhage was followed by a copious serous exudation.

The attenuated portion of the integument had now become mottled and livid. In some places exudations would take place which would discharge a sero-sanguinolent fluid ; at others, large patches of skin would vesicate, which, when ruptured, would discharge large quantities of *sero-purulent* fluid. Unlike the sanguineous discharges, the serous, when profuse, would very materially lessen the size of the tumor. These effusions now became very copious, excoriation very extensive, and finally the whole mass became succulent and loathsome, and on the twelfth day the unfortunate being died.

It is to be very deeply regretted that circumstances prevented an autopsic examination, which would have relieved us of the necessity of making a conjectural diagnosis. When we take into consideration the peculiar form of the cranium, the exacerbations of chills and fever, the suppression of the secretions, the spasmodic and paralytic symptoms, the pedunculated form of the tumor, its situation, the fluctuation, it would seem to be a well marked case of *hydrencephalocele.* Unfortunately, however, this conclusion would be successfully controverted by a fact which was easily demonstrated, *i. e.*, that there existed no opening in the bone through which such a hernia could protrude. As before stated, the posterior fontenelle, and the entire surface of the occipital bone having any relation whatever to the cephalocele, was closely examined and with positive results. Discarding, then, the idea of *hernia meningea, or encephalocele,* we still have a dropsical tumor, as is clearly shown by the fluctuation, its being limited in its compressibility, its being most fluctuating when most compressed, by the nature and quantity of the effusions, etc. And not only this, but there is abundant evidence to show that it was a venous erectile tumor, as well. Witness, for example, its compressibility, its pulsation, the venous murmurs, the effect of long continued compression of the carotids, the effect of different states of the circulation, for example, determination, the hemorrhage, the plexuses of veins that were clearly recognizable, etc. We think ourselves justified in attributing to the tumor the two pathological elements above mentioned, from all the rational and objective symptoms to be derived from the tumor itself. But what are we to say of the paralysis, the convulsive and spasmodic action, the coldness and blueness of the left side, and the state of the organic functions? We have but one resort, *i. e.*, if the tumor had no connection with the nervous centres, it exercised the strongest possible sympathetic sway over both the cerebro-spinal and sympathetic systems.

Concerning its cause, perhaps some might be curious to know that those matrons who are reputed to have founded medical science, marvellously elucidated the etiology of this affection in this wise: When Mrs. S. was one month advanced in gestation, her father-in-law was confined to bed with double inguinal hernia. Having an urgent call to defecate, he jumped out of bed in her presence, when by accident the distended scrotum was observed, to *her* great terror and dismay, and to the repetition of a similar deformity about the fœtus, *perhaps erroneously* upon the head, and most certainly so, in that the fissure was transverse.

It required no great stretch of their imaginations to suppose this idea absolutely confirmed, by the presence of the fissure, which they supposed to be the septum. By the way, physical examination proved this septum to be merely apparent.

I do not feel warranted in discarding this explanation, however, merely from the singularity of the site of the tumor, as there are other cases on record of similar coincidences. An account of a congenital cephalocele, occurring on a boy of 11 years, is recorded by Mr. Hosmer in the Boston Med. and Surg. Journ. for Nov., 1855, in which the following cause was assigned by the mother. "While she was pregnant, a large hog, which had recently been emasculated, passed frequently by her house. *The appearance of the animal produced an unpleasant sensation in her, which caused her always to place her hand upon her head.*" From the brief history of this case, I should judge it to be one of *encephalocele.* These cases (though dissimilar in that one tumor contained brain, and the other none,) might by some be made parallel, by taking into consideration the different states of the objects of aversion. Or, perhaps, the reverse view would be entertained by some of the great psychological lights of our age,

*i. e.*, to suppose the tumors to be real and imaginary phrenological elongations of the cerebellum, which would be in keeping with phrenological demonstration, *i. e.*, by coincidences.

